# Various arrangements of mobile genetic elements among CC147 subpopulations of *Klebsiella pneumoniae* harboring *bla*_NDM-1_: a comparative genomic analysis of carbapenem resistant strains

**DOI:** 10.1186/s12929-023-00960-0

**Published:** 2023-08-25

**Authors:** Omid Pajand, Hamzeh Rahimi, Farzad Badmasti, Faeze Gholami, Tahereh Alipour, Narges Darabi, Frank M. Aarestrup, Pimlapas Leekitcharoenphon

**Affiliations:** 1 Research Group for Genomic Epidemiology, National Food Institute, Technical University of Denmark, Lyngby, Denmark; 2 Clinical Research Development Unit, Kowsar Educational, Research and Therapeutic Hospital, Semnan University of Medical Sciences, Semnan, Iran; 3 Abnormal Uterine Bleeding Research Center, Semnan University of Medical Sciences, Semnan, Iran; 4 Department of Bacteriology, Pasteur Institute of Iran, Tehran, Iran; 5 Social Determinants of Health Research Center, Semnan University of Medical Sciences, Semnan, Iran; 6 Microbiology Department, Faculty of Medicine, Semnan University of Medical Sciences, Semnan, Iran

**Keywords:** *Klebsiella pneumoniae*, Iran, MGEs, OXA-48, High-risk clones, ST147, *armA*, Whole genome sequencing, Phylogeny

## Abstract

**Background:**

Certain clonal complexes (CCs) of *Klebsiella pneumoniae* such as CC147 (ST147 and ST392) are major drivers of *bla*_NDM_ dissemination across the world. ST147 has repeatedly reported from our geographical region, but its population dynamics and evolutionary trajectories need to be further studied.

**Methods:**

Comparative genomic analysis of 51 carbapenem-nonsusceptible strains as well as three hypervirulent *K. pneumoniae* (hvKp) recovered during 16-months of surveillance was performed using various bioinformatics tools. We investigated the genetic proximity of our ST147 strains with publicly available corresponding genomes deposited globally and from neighbor countries in our geographic region.

**Results:**

While IncL/M plasmid harboring *bla*_OXA-48_ was distributed among divergent clones, *bla*_NDM-1_ was circulated by twenty of the 25 CC147 dominant clone and were mostly recovered from the ICU. The NDM-1 core structure was bracketed by a single isoform of mobile genetic elements (MGEs) [ΔIS*Kpn26*-NDM-Tn*As3*-ΔIS*3000*-Tn*5403*] and was located on Col440I plasmid in 68.7% of ST392. However, various arrangements of MGEs including MITE*Sen1*/MITE*Sen1* composite transposon or combination of MITE*Sen1*/IS*Sen4/*IS*903B/*IS*5/*IS*Ehe3* on IncFIb (pB171) were identified in ST147. It seems that ST392 circulated *bla*_NDM-1_ in 2018 before being gradually replaced by ST147 from the middle to the end of sample collection in 2019. ST147 strains possessed the highest number of resistance markers and showed high genetic similarity with four public genomes that harbored *bla*_NDM-1_ on the same replicon type. Mainly, there was a convergence between clusters and isolated neighboring countries in the minimum-spanning tree. A conserved arrangement of resistance markers/MGEs was linked to methyltransferase *armA* which was embedded in class 1 integron in 8 isolates of ST147/ST48 high-risk clones.

**Conclusion:**

Our findings highlight the dynamic nature of *bla*_NDM-1_ transmission among *K. pneumoniae* in Iran that occurs both clonally and horizontally via various combinations of MGEs. This is the first analysis of Iranian ST147/NDM + clone in the global context.

**Supplementary Information:**

The online version contains supplementary material available at 10.1186/s12929-023-00960-0.

## Introduction

*Klebsiella pneumoniae* is a member of the *Enterobacterales* family with an increasing rate of antimicrobial resistance, owing primarily to the production of carbapenemases and extended-spectrum beta-lactamases (ESBLs). This has led to the “Centers for Disease Control and Prevention” (CDC) to classify this organism as an urgent threat [1]. In addition, this bacterium is classified as a “priority pathogen” by “surveillance and infection control agencies” [2]. Molecular analyses have shown that the spread of epidemic lineages (high-risk clones) such as clonal complexes CC15, CC101, CC147, and CC258 in different geographical regions promotes the increasing emergence of multidrug resistant (MDR) *K. pneumoniae* strains [3–5].

Global healthcare systems have experienced an increase in the emergence and spread of epidemic lineages of carbapenem resistant *K. pneumoniae* (CRKp) since 2001 [6, 7]. The CC147, including sequence types (ST) ST147 and ST392, has been identified as a successful high-risk clone in the spread of carbapenemases [8]. In the years from 2010 to 2014, the ST147 clone was identified as a vehicle for the distribution of *bla*_NDM_ worldwide using various mobile genetic elements (MGEs), including IncX3, IncFIIA and IncA/C plasmid types [9]. According to the antimicrobial resistance reports of the “Center for Disease Dynamics, Economics, and Policy”, Iran had a trend of 43–45% of CRKp during 2018 and 2019 [10]. While the emergence of *bla*_NDM_ has been described in Iran since 2013 [11], a deep genomic study based on whole genome sequencing (WGS) has been published recently [12]. Sequence types ST11, ST268, ST893, and CC147 have been linked to *bla*_NDM_ and *bla*_OXA-48_ distributed across the country, with the latter being common in different surveys [13, 14]. In view of the relatively widespread distribution of CRKP in Iran [15], it is necessary to analyze the population structure and resistance/virulence mechanisms of these strains. By combining new tools like WGS with information from epidemiological, clinical, and phenotypic sources, we can learn more about how bacteria spread, how dangerous they are, and how antimicrobials affect them.

In follow-up to our previous cross-sectional study describing the occurrence of CC147 strains co-harboring *bla*_NDM_/*bla*_OXA-48_ at Semnan province [16], carbapenem non-susceptible *K. pneumoniae* strains were subjected to WGS. The aim of this study was to focus on the CC147 subpopulation and mostly the ST147 in the context of a local hospital and on a worldwide scale to gain an understanding of the evolution, genomic content, and plasmid repertoire contributing to success of this high-risk clone.

## Materials and methods

### Collection of bacterial population and antibiotic susceptibility patterns

The study was conducted in a single acute-care referral hospital in Semnan, Iran, that covered > 120,000 people in this province. During the 16-month surveillance study from March 2018 to June 2019, out of 123 *Klebsiella* spp. isolates were recovered from extra-intestinal specimens. Isolates were phenotypically identified by standard biochemical tests, including the sugar fermentation pattern on Triple Sugar Iron Agar, the pattern of SH2/Indole/Motility reactions on SIM, Citrate consumption, and Urease production [17].

The disk diffusion test was applied for susceptibility testing of collected isolates, and the results were interpreted based on the Clinical and Laboratory Standard Institute (CLSI) guidelines [18]. A resistant phenotype against one of the three or more antimicrobial classes was considered for the multidrug resistant (MDR) definition [19]. Strains showing a non-susceptible phenotype against either of the study carbapenems (imipenem, meropenem, or ertapenem) were considered carbapenem non-susceptible and subjected to minimum inhibitory concentration (MIC) determination using E-test strips. The MIC criteria for considering of susceptibility against imipenem/meropenem and ertapenem were ≤ 1 μg/ml and ≤ 0.5 μg/ml, respectively [18].

### Analyses of sequencing data

The first step of whole genome sequencing analysis was DNA extraction and an Invitrogen Easy-DNA kit was used for this purpose. After determination of DNA concentration using the Qubit dsDNA BR assay kit (Invitrogen), library preparation was conducted according to the Illumina protocol and sequencing was run on NextSeq and MiSeq platforms using 150 bp paired-end reads [20].

Evaluation of raw read quality was done by FastQC version 0.11.5 (https://www.bioinformatics.babraham.ac.uk/projects/fastqc/), and TrimmomaticPE version 0.22 [21] was used for trimming with the following parameters: The minimum quality required for maintaining a baseline from the beginning and from the end of the read was Q30, and the average quality was based on a window size of 10 with Q20. De novo assembling of raw reads was performed by SPAdes version 3.11.0 [20]. The quality evaluation of generated assemblies was done with QUAST version 4.6.3 [22]. In silico bioinformatics tools were used for analyzing assembled sequences in order to confirm the sequence types (STs), plasmid replicons, acquired resistance markers, fluoroquinolone resistance associated mutations, and plasmid MLST (pMLST) using the following pipelines: MLST 2.0 [23], plasmidFinder 2.1 [24], ResFinder 4.1 [25], and pMLST 2.0 [23]. Single target analyses using the NCBI BLAST tool were performed with the BIGSdb-Kp database curated by the Pasteur Institute (https://bigsdb.pasteur.fr/). Genomes were uploaded into the Pathogenwatch global platform in order to obtain information on capsular and O-antigen serotyping, and Integrative Conjugative Elements (ICEs) harboring aerobactin/salmochelin/yersiniabactins (https://cgps.gitbook.io/pathogenwatch/). The annotation of some related resistance carrying contigs was performed by the RAST online server and, gbk format files were downloaded. The MGEFinder 1.0 was used to find the association of resistance markers with MGEs [26], and the origin of chromosome, phage, and plasmid derived sequences was detected by SourceFinder 1.0 [27]. The Geneious Prime 2021.1.1 was applied for graphical annotation of genetic environment of contigs harboring resistance genes and the position of insertion sequences on these contigs was manually curated using ISFinder BLAST results [28, 29]. The similarity determination of genomes to hvKp virulence content was done by using the Blast Ring Image Generator (BRIG) [30].

We also studied the ST147 strains in a global context. To do this, the raw-fastq.gz reads and assemblies were downloaded from public databases and subjected to two different platforms. First, the ST147/NDM + raw reads (n = 128) from the United States that were recently deposited in the European Nucleotide Archive (ENA) were downloaded [31]. Furthermore, 46 ST147/NDM + raw reads from different studies of Europe, Southeast Asia, South-America, and Canada were also included [32]. Totally, 174 ST147/NDM + short read data sets were subjected to the pipelines mentioned above and the CSI phylogeny (as described below) to draw a SNP-phylogenetic tree. Second, cgMLST analysis was used to study our ST147 strains and corresponding genomes (both *bla*_NDM_ + ve or − ve) deposited from countries in our geographic region (n = 121) by downloading assemblies from the PATRIC database (https://www.patricbrc.org/, accessed on 29 June 2023). To access these genomes, filters such as ‘Iran’, ‘Pakistan, ‘India’, ‘Turkey’, ‘United Arab Emirates’ (UAE), ‘Russia’, ‘Lebanon’ and ‘Israel’, ‘MLST: 147’, ‘genome quality: good’ were used. The FASTA format of pre-assembled sequences was retrieved to subject Ridom SeqSphere + software to drawing the Minimum Spanning Tree (MST) (described below). Other information, such as the collection year and sample origin for each genome, was collected from PATRIC.

Reference-based analysis was used to draw the phylogenetic tree using reference strain *K. pneumoniae* strain 4/1–2 (GenBank ID: Cp023839.1). The CSI Phylogeny 1.4 was used to draw the SNP tree [33]. The Burrows-Wheeler Aligner (BWA), version 0.7.2 was used to map of paired end reads to the reference genomes and the mpileup module in SAMTool version 0.1.18 was used for SNP calling [34]. The criteria that were considered for selection of SNP were: (i) presence of minimum 15bp distance between each SNP (pruning), (ii) minimum 10% of average depth, (iii) mapping quality > 30, (iv) quality of SNP > 20, and (v) all indels were excluded. The quality passed SNPs of each genome were concatenated into a single alignment corresponding to the position of the reference strain. The concatenated sequences were subjected to phyML [35] for the construction of a parsimony tree by using the HKY85 substitution model and 1000 bootstrap replicates. The pairwise 23 SNP distances between isolates proposed by Sherry et al. were used for the definition of local transmission [36]. The web interface software iTol was used to visualization and labeling of the generated phylogenetic tree. By putting the assembled genomes on Ridom SeqSphere (Ridom GmbH, Munster, Germany) [37], the cgMLST allelic mismatch between strains was used to make the MST. According to the cgMLST SeqSphere + server, closely related genomes are ‘lumped’ together in complex types (CTs; https://www.ridom.de/u/Core_Genome_MLST_Complex_Type.html) and first approximation of close genetic relatedness is defined based on a CT distance of 15 allele differences (https://www.cgmlst.org/ncs/schema/2187931/). This CT allele distance threshold is based on retrospective analysis of well-defined outbreaks and outgroup isolates with the same MLST/MLVA/PFGE profiles, as described elsewhere [38]. Moreover, data regarding the virulence factors and resistance genes content of downloaded assemblies was obtained by running the “*K. pneumoniae* VFDB” and “NCBI AMRFinderPlus” pipelines implemented in Ridom SeqSphere + software.

### Transconjugation assay

A filter-mating experiment was performed to evaluate the horizontal gene transfer (HGT) potential of plasmids [39]. Three strains (two ST392 and one ST147, all *bla*_OXA-48/NDM-1_ +) were considered as potential donors. A colony of *K. pneumoniae* strains was used to inoculate LB broth (5 ml). In addition, a colony of *Escherichia coli* DH5α was also used to inoculate LB (5 ml) as recipient. After overnight incubation, the cultures were washed twice with PBS and then mixed at a ratio of 10:1 *K. pneumoniae* (donor) to *E. coli* (recipient) in 300 µl PBS. The mixture was plated on a 0.22 µm pore size membrane filter on LB agar and incubated overnight. The cells on the membrane were then re-suspended in 10 ml of PBS and plated on MacConkey agar supplemented with both nalidixic acid (32 µg/ml) and cefotaxime (1 µg/ml). Pink colonies (as recipients) were purified. PCR assays were used to confirm the presence of *bla*_NDM-1_ and *bla*_OXA-48_ in transconjugants [39].

### Statistical methods

Statistical analysis was done using IBM-SPSS Statistics 22. Bivariate analyses of categorical variables were done by Chi-square test. All *P* values were two-sided.

## Results

### Patients’ characteristics

Our study enrolled all culture-positive plates of Gram-negative bacteria recovered from extraintestinal infections during a 16-month period. A total of 123 *Klebsiella* spp. were identified through biochemical testing among these consecutive samples. Of those, 51 patients (53.7% female, 46.3% male, age range was from 10 to 93 years) with a clinical history consistent with carbapenem non-susceptible (including either imipenem, meropenem or ertapenem disk diffusion test results) *K. pneumoniae* infections were subjected for further analyses. In addition, three patients infected with carbapenem-susceptible hypervirulent *K. pneumoniae* were included for comparative process. The majority of the isolates were from urine cultures (29, 53.7%), respiratory (20, 37%), wound (2, 3.7%), and blood (2, 3.7%). Patients were admitted or stayed at four distinct wards; Intensive Care Unit (ICU) (66.7%), Internal (22.2%), Cardiac Care Units (CCU) (3.7%) and Emergency/observation service (7.5%).

### Amikacin was the most potent antibiotic against carbapenem non-susceptible *K. pneumoniae*

Resistance rates against imipenem, meropenem, ertapenem, cefepime, and trimethoprim/sulfamethoxazole antibiotics were 75.9%, 70.4%, 83.3%, 72.2%, and 72.2%, respectively. Isolates were highly resistant against ampicillin/sulbactam and amoxicillin/clavulanic acid (88.9% resistant rates for both), and piperacillin/tazobactam and aztreonam (83.3% resistance rates for both). Amikacin was figured out as the most potent antibiotic with a 22.2% resistance rate, followed by tobramycin (63%) gentamicin (64.8%), and levofloxacin (66.7%). The MDR phenotype was found in 87% of the isolates tested. The MICs of carbapenems against *bla*_OXA-48_ and *bla*_NDM-1_ producers are shown in Table 1.

**Table 1:** Demographic characteristics of *K. pneumoniae* subjected to WGS

Strain No	ST^a^	KL, O-type^b^	Ward of isolation	Sample^c^	MDR ^d^	age	Gender ^e^	Yersiniabactin; ICE	IMI, MEM, ETP ^f^ (μg/ml)
35	14	16, O1v1	Internal ICU	Respiratory	**+ **	85	F	*ybt* 1-; ICE*Kp*4	
56^♦^			Internal ICU	UC		68	F		2, 2, 32
361^♦^			Internal ICU	UC		68	F		0.5, 1, 2
364^♦^			Internal	UC		83	F		0.5, 0.38, 2
607	40	155, OL101	Internal	UC	**+ **	10	F	Negative	1, 1, 32
635			Internal	Respiratory		74	F		
691			Surgical ICU	UC		14	F		
152^♦^	45	52, OL101	Internal ICU	BC	**+ **	54	F	*ybt* 1-; ICE*Kp*4	6, 6, 32
190^♦^			Internal ICU	Respiratory		89	M		0.5, 0.38, 0.75
200^♦^			Internal ICU	Respiratory		85	M		1.5, 2, > 32
525^♦^			CCU	Respiratory		93	F		2, 1, > 32
531^♦^			Internal ICU	Respiratory		79	M		1, 0.75, 16
606^♦^	48	2, O1v1	Internal	UC	**+ **	79	M	Negative	1, 1, > 32
639^♦^			Internal ICU	Respiratory		83	F		> 32 to all
689^♦^			Internal ICU	Respiratory		82	F	*ybt* 1-; ICE*Kp*4	0.5, 0.5, 1
693^♦^			Internal ICU	Respiratory		80	F		4, 4, 32
290	86	2, O1v1	OPD	UC	−	64	F	*ybt* 9; ICE*Kp*3	
533			Emergency	UC		64	F		
185*	147	64, O2v1	Surgical ICU	UC	**+ **	80	M	Negative	> 32 to all
500*			Internal ICU	Respiratory		67	F		
558*			Internal ICU	UC		90	M		
589*			Internal ICU	Respiratory		85	M		
640*			Internal ICU	UC		79	F		
653*			Surgical ICU	Respiratory		76	M		
655*^♦^			Internal	UC		54	F		
697*			Surgical ICU	UC		72	M		
718*			Internal ICU	BC		65	F		
254	256	47, OL101	Surgical ICU	BC	**−**	61	M	Negative	
298	348	62, O1v1	OPD	UC	**−**	90	F	*ybt* 14; ICE*Kp*5	
560^♦^	377	102, O2v2	Internal ICU	Respiratory	**+ **	34	M	*ybt* 16; ICE*Kp*12	> 32 to all
686^♦^			Internal ICU	Respiratory		54	M		4, 4, 16
111*	392	27, O4	Surgical ICU	Respiratory	**+ **	33	M	*ybt* 9; ICE*Kp*3	> 32 to all
117*^♦^			Surgical ICU	Respiratory		35	M		> 32 to all
2*			Internal ICU	Respiratory		68	F		6, 16, 32
275*^♦^			Internal ICU	UC		83	F		3, 24, 24
432*			Internal ICU	Respiratory		77	F		6, 6, 16
350^♦^			Internal ICU	Respiratory		85	M		1.5, 4, 32
351^♦^			Internal	Respiratory		76	M		0.5, 0.5, 32
191*^♦^			Surgical ICU	UC		49	M		24, 6, 32
247*^♦^			Internal ICU	UC		89	M		12, 6, 16
118*^♦^			Internal	UC		54	F	Negative	32, 12, 32
434*			Internal ICU	Wound		26	M		6, 6, 8
447*			CCU	UC		63	M		> 32 to all
45^♦^			Internal ICU	Wound		82	F		12, 12, 32
460			Surgical ICU	UC		90	M		ND
659*			Internal	UC		89	M		> 32 to all
77^♦^			Internal ICU	UC		34	F		4, 4, 32
700	815	107, O4	Internal ICU	UC	**−**	65	F	Negative	
416	1308	144, O4	OPD	UC	**+ **	27	M	Negative	
670		144, O4	Internal	UC	**+ **	32	F		
671		144, O4	Internal	UC	**+ **	61	M		
114	1565	31, OL104	Internal	UC	**−**	83	M	Negative	
542	2159	1, O1v2	Surgical ICU	UC	−	71	F	Negative	
22	6510	22, O2v1	Internal	UC	**+ **	85	F	Negative	

^♦^Indicates carrying *bla*_OXA-48_, *Indicates carrying *bla*_NDM-1_, a: Sequence type; b: capsular type; c: BC & UC stand for blood and Urine culture, respectively; d: Multidrug resistant; e: Female, Male; f: Minimum Inhibitory Concentrations of imipenem, meropenem and ertapenem are determined for some of the study strains

### CC147 was detected as the dominant clone

The study isolates were categorized into 15 STs and CC147 (including ST147 [9 strains] and its single locus variant (SLV) ST392 [16 strains]), was identified as the dominant clone, accounting for 25 (46.2%) episodes of infection. The other prevalent clones were ST45 (9.2%), and two high-risk clones, ST14 and ST48 (7.4%, for each).

**Fig. 1:**
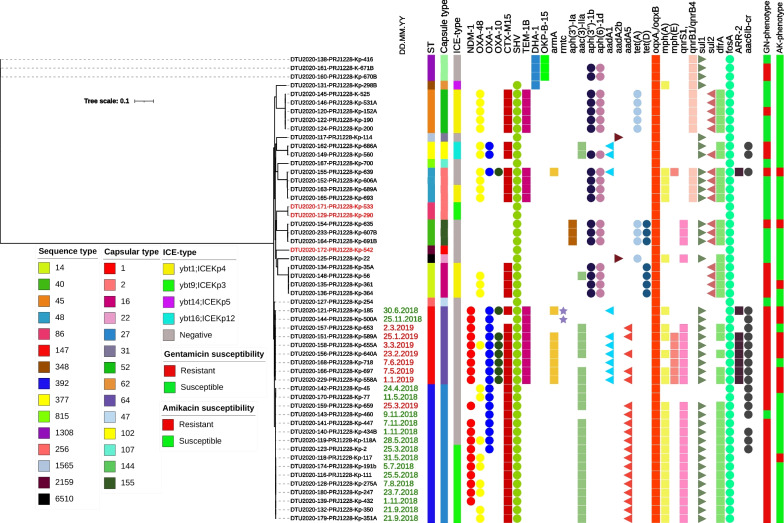
Phylogenetic tree of study 54 *K. pneumoniae* isolates. Maximum-likelihood phylogenic tree clustered different clones (STs) and CC147 strains (ST147, ST392) were identified as the dominant clone with the highest prevalence of varied resistance elements. The tree was constructed from the whole genome SNPs arising by mutation and labeling and visualization were done by using the iTOL web-interface. The data presented are the date of isolation for CC147, sequence types, capsular types, Integrative and conjugative Elements (ICEs), acquired resistance markers and amikacin/gentamicin susceptibility phenotypes. The hvKp strains have been shown in red

Average nucleotide identity (ANI), with ANI values obtained for each species exceeding 97%, supported clustering by drawing the phylogenetic tree. Based on phylogenetic analysis of 88,634 genome-wide SNPs, strains were divided into *K. pneumoniae* KpI (51, 94.4%) and KpII subgroups of *K. quasipneumoniae* subsp. *quasipneumoniae* KpII-A (3, 5.6%) that belonged to ST1308 (Fig. 1). The distribution of capsular locus (KL) types across the phylogenetic tree revealed their highly clonal nature, with each KL type observed only in one ST, except for KL-2 (O locus; O1v1), which was shared by ST48 and two hvKp ST86 strains. We identified 14 different K loci and 5 O loci, and these K/O loci provided 14 different combinations in our collections. The most frequently identified KL types were KL-27 and KL-64 which were linked to ST392 and ST147, respectively (Table 1). The O1/O2v1 loci were the most prevalent O antigens, detected in 22 (40.7%) isolates of different STs including ST14, ST48, ST86, ST147, ST348, ST2159 and ST6510. The O4 serotype was the second most common O locus and was identified in 20 strains of ST392, ST815, and ST1308. We observed the same O-Locus combined with a distinct K-Locus, i.e., O1v1 was associated with KL-2/KL-16/KL-62, O2v1 in KL-64/KL22, O101 was associated with KL-155/KL-52 /KL-47, and O4 was linked to KL-144/KL-27/KL-107 (Table 1).

The majority of patients from whom CC147 strains recovered were admitted to an intensive care unit (internal ICU, surgical ICU), where strains were isolated during their stay. Besides the ICU ward, one of the CC147 infection episodes was recovered from the urine culture of a patient admitted to the CCU (strain no. 447, ST392). Temporally, the first NDM positive strain (strain no. 2, ST392) was isolated shortly after starting of sample collection (March 2018), and all except one ST392 strain (strain no. 659) were recovered during 2018. In contrast, ST147 appeared in June 2018 and replaced ST392 during the entire sampling period (Fig. 1). In terms of carbapenem MICs, the ST147 clone did better than the ST392/NDM + strains against this family of antibiotics (Table 1).

To identify genomic clusters that are likely to be epidemiologically linked, 23 SNP pairs were used as a pairwise distance cutoff. The ST147 strains were clustered as the significant phylogroup with limited SNP (5–19) divergence, suggesting an outbreak of this clone in our hospital. In contrast, ST392 was relatively heterogeneous, with more detectable SNPs (2–48), indicating lower clonality in this variant.

**Fig. 2:**
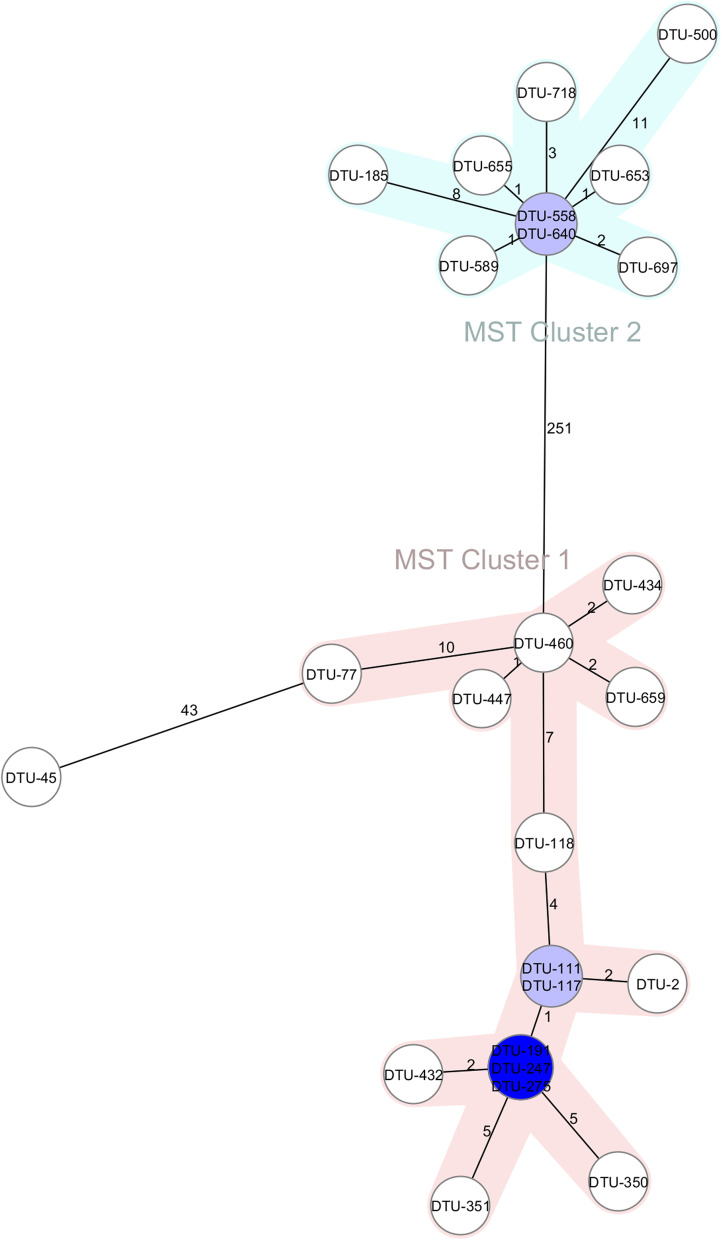
Minimum spanning tree of CC147 based on the core genome MLST. The 25 CC147 strains were analyzed based on comparing 2365 alleles calculated in Ridom SeqSphere. Cluster was defined based on the maximum allelic distance of ≤ 15 alleles. Inside the bubbles the isolate ID numbers are shown, and the allelic distances between isolates are represented on the lines connecting them. Clusters 1 (ST392) and 2 (ST147) are shown by pink and pale blue zones, respectively

Clustering of CC147 genomes based on a minimum spanning tree revealed a high level of proximity among ST147 strains (maximum of 11 allelic distances), while ST392 exhibited shallow branching and one strain (No. 45) (OXA-48^+^/NDM^−^) had a relatively high allelic distance (43 allele differences) from the nearest ST392 node (Fig. 2).

### The highest prevalence of resistance markers detected in ST147

Except for *fosA* and *oqxA*/*oqxB* (efflux pumps), which were detected in all study populations, the most common resistance marker was *bla*_SHV-_ (51, 94.4%), which was chromosomally located in all positive strains, followed by *bla*_CTX-M-15_ (39, 72.2%), *dfrA* (38, 70.4%), *sul1* (37, 68.5%), *aac3IIa* (27, 50%), *qnrS* (25, 46.3.1%), *bla*_OXA-1_ (20, 37.03%) and *aac6-Ib-cr* /*aph(3´)-Ib* / *aph(6´)-Id / aadA5* (19, 35.1% for each).

The *bla*_OXA-48_, *bla*_NDM-1_ and *bla*_OXA-48/NDM-1_ were detected in 18 (33.3%), 14 (24.9%), and 6 (11.1%) strains, respectively. While *bla*_OXA-48_ was distributed among different STs including ST14, ST45, ST48, CC147, and ST377, the dual carbapenemases of *bla*_OXA-48_/*bla*_NDM-1_ was detected in CC147 and mostly in ST392 (5 strains). Focusing on the CC147 resistance gene content revealed that this clonal group harbored a significantly higher number of different resistance markers compared to other STs. However, it wasn’t uniform when the two members were considered separately. It means ST147 had a more homogeneous combination of resistance markers than ST392, including *bla*_NDM-1_ (9 [100%] *vs*. 11 [68.8%], *P*: 0.1), *sul1* (9 [100%] *vs*. 14 [87.5%], *P*: 0.02), *bla*_OXA-1_ (9 [100%] *vs*. 8 [50%], *P*: 0.02), *bla*_OXA-10_ (7 [77.8%] *vs*. 0, *P* < 0.001), *armA* (7 [77.8] % *vs*. 0, *P* < 0.001), *aac(6´)-Ib-cr* (9 [100%] *vs*. 7 [43.8%], *P*: 0.008) and *bla*_TEM-1B_ (9, [100%] *vs*. 0, *P* < 0.001). The exception was *bla*_OXA-48_ which was markedly harbored by ST392 (9 [56.2%], *P*: 0.04) (Fig. 1). Furthermore, mutations in the OmpK36 porin (Gly134Asp135 duplication in loop 3, OmpK36GD) that constrict this porin channel and render it resistant to antibiotics were found in all ST147 and two ST377 strains.

The association between carrying resistance elements and resistant phenotypes was assessed. Isolates harboring *bla*_OXA-1_ were significantly resistant to aztreonam (*P*: 0.01) and cefepime (*P* < 0.001). Moreover, the *bla*_CTX-M-15_ carriage was in association with a resistant phenotype to aztreonam, piperacillin/tazobactam, ampicillin/sulbactam, ceftazidime, cefepime, amoxicillin/clavulanate (*P* < 0.001 for all), and cefotaxime (*P*: 0.01). Resistance against amikacin was associated with the carriage of *aac(6´)-Ib-cr* (9 [75%], *P*: 0.002) and *armA* (7 [58.3%], *P* < 0.001), while gentamicin resistance was detected in strains harboring *aac(6´)-Ib-cr* (18 [51.4%], *P*: 0.001), *aac3Iia* (27 [77.1%], *P* < 0.001), and *armA* (8 [22.9%], *P*: 0.04). The least frequent resistance marker was the 16S rRNA methylase gene *rmtC* which harbored by two ST147 strains, both were resistant against all aminoglycosides. The aminoglycoside nucleotidyltransferase *aadA5* was detected in 18 CC147 strains and was in strong association with resistant phenotype against gentamicin (18 [94.7%], *P*: 0.001) and tobramycin (19 [100%], *P* < 0.001). Even when the nucleotidyl-phosphatases *aph(6’)-Id* and *aph(3’)-Ib* were combined, resistance to the three aminoglycosides tested was not associated (Table 2). The proportions of acquired resistance markers which provide resistant phenotypes against the study antibiotics have been shown in sunburst plot (Additional file 1).

**Table 2:** Resistance genotypes in association with phenotypic characteristics

Resistant phenotype	*gyrA*/*parC n (%)*^***a***^	*qnrS1*	*aac(6)-Ib-cr*	*aadA1*	*aadA5*	*armA*	*aac3IIa*	*aph3Ib/aph6Id*
Ciprofloxacin (n = 41)	27 (65.9%)	25 (61%)	19 (46.3%)	ND	ND	ND	ND	ND
*P* values	**0.001**	** < 0.001**	**0.002**	–	–	–	–	–
Levofloxacin (n = 36)	27 (77.1%)	23 (65.7%)	19 (54.3%)	ND	ND	ND	ND	ND
*P* values	**< 0.001**	**< 0.001**	**< 0.001**	–	–	–	–	–
Amikacin (n = 12)	ND	ND	9 (75%)	7 (58.3%)	5 (41.7%)	7 (58.3%)	8 (66.7%)	3 (25%)
*P* value	–	–	**0.002**	**< 0.001**	0.9	**< 0.001**	0.3	0.5
Gentamicin (n = 35)	ND	ND	18 (51.4%)	10 (28.6%)	18 (51.4%)	8 (22.9%)	27 (77.1%)	10 (28.6%)
*P* value	–	–	**0.001**	0.01	**0.001**	**0.04**	** < 0.001**	0.2
Tobramycin (n = 34)	ND	ND	19 (55.9%)	10 (29.4%)	19 (55.9%)	8 (23.5%)	27 (79.4%)	8 (23.5%)
*P* value	–	–	**< 0.001**	**0.009**	**< 0.001**	**0.02**	**< 0.001**	0.03

a: n (%) represents the positivity within resistance rates against each antibiotic

ND: not determined

Underlined: represents negative association

Bolded: represents the positive association

The fluoroquinolone-resistant (FQ-R) phenotype (non-susceptibility to either ciprofloxacin or levofloxacin) was found in 42 (77.7%) of the study isolates and was associated with *parC* (S80I) + *gyrA* (S83I) mutations (27 [64.3%], *P*: 0.001) or carrying the *qnrS1* gene (25 [59.5%], *P*: 0.001) and *aac(6´)-Ib-cr* (19 [45.2%], *P*: 0.004). Among the CC147, all were concomitantly positive for *qnrS1* and *gyrA* S83I / *ParC* S80I, except two ST147 and two ST392 strains, which were negative for *qnrS1*. The aforementioned genotypes were mostly prevalent in CC147 strains. The other PMQR found was *qnrB1*/*B4*, which was detected in 13 ST45, ST48, ST348, and ST1308 strains but was not linked to fluoroquinolone resistance (Table 2).

### Different plasmid repertoires were found in CC147 members

**Fig. 3:**
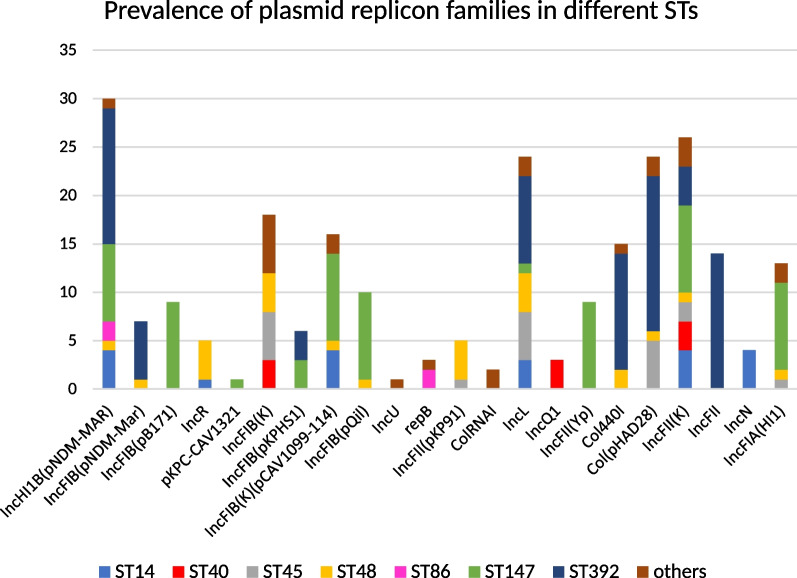
Bar-plot displaying the prevalence of different replicon types and their association with STs. The Y-axis represents the number of positive isolates in each STs

Concerning plasmid replicon types, a total of 23 replicon types were found. The IncF was the dominant plasmid type that was identified in all isolates, followed by the IncHI1B (31, 57.4%), IncL (24, 44.4%), and the col_pHAD28 (24, 44.4%). The most common IncF-type plasmids were IncFII_K (26, 48.1%), IncFIB_K (18, 33.3%), and IncFIB(K) (pCAV1099-114) (16, 29.6%). Furthermore, the two members of CC147 showed different patterns of replicon types. The IncFIA (HI1), IncFII_K, IncFII_Yp, IncFIB (pQil), IncFIB (K) (pCAV 1099-114), and IncFIB (pB171) (*P*: 0.001 for all) were detected significantly in ST147, and the IncHI1B (pNDM-MAR), IncL (pHAD28), Col440I, and IncFII were remarkably harbored by ST392. The three types of plasmids, IncFII(Yp), IncFIB (pB171), and pKPC-CAV 1321, were exclusively detected in ST147 strains (Fig. 3).

On three occasions, it was possible to link replicon type and resistance elements to the same contig: (i) ST147 strains carried *bla*_NDM-1_ on an IncFIB (pB171). (ii) ST392 harboured *bla*_NDM-1_ on a Col440I, and (iii) *bla*_OXA-48_ was embedded in a Tn*1999* composite transposon in association with the IncL/M replicon type, the notorious plasmid lineage responsible for the worldwide dissemination of *bla*_OXA-48_.

### The *bla*_NDM-1_^+^ transconjugants was successfully developed

By using conjugation experiment, the NDM-1^+^ plasmids of 3 studied isolates (ST147 and 2 ST392 strains) were successfully transferred to recipient, conferring resistance to carbapenems and cephalosporins in transconjugants. Plasmid gel extraction and the following PCR experiment of the transconjugants revealed that the *bla*_NDM-1_ was located on conjugative plasmids. However, in the experiments the OXA-48^+^ transconjugants were not detected.

### Clones differed in their content of virulence genes

We analyzed additional important virulence factors such as yersiniabactin, aerobactin, and salmochelin siderophore systems in the study population, whose bacterial survival has been shown to be enhanced by acquisition of iron from the host. Aerobactin *iutA* and capsule *wzi* were the most commonly identified virulence factors, and they were found in all isolates. Type 1 (*fim*) and type 3 (*mrk*) fimbriae gene clusters are major adhesins to biotic and abiotic surfaces. Type 1 gene cluster was identified in all isolates. The “mannose-resistant *Klebsiella*-like (type 3) fimbriae cluster” (*mrkABCDFHIJ*) was detected in 47 (87%) of our collection.

**Fig. 4:**
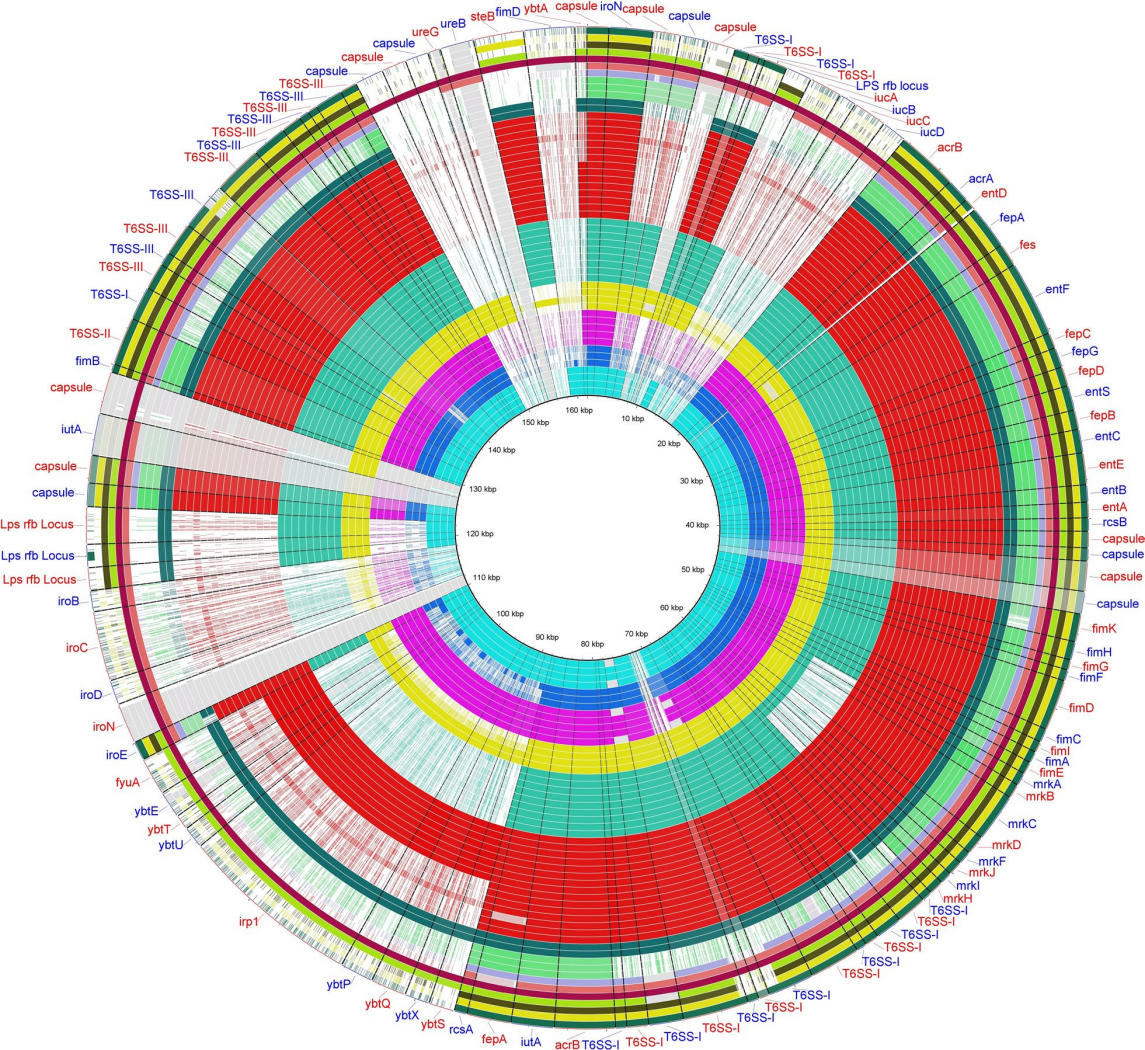
The virulence genes content of *K. pneumoniae*. A BLAST Ring Image Generator (BRIG) image shows the presence of virulence factors among different STs. The FFN format file of hvKp (strain No. 290 identified as ST86) was downloaded from VFDB website, and open reading frames (ORFs) are annotated based on the VFDB results and used as reference strains to draw the image. Aligning between the regions of interest and each genome are shown as Colored segments (indicate > 70% similarity), and gray segments (indicate > 50% similarity). From inside to outside of the figure: “deep sky blue “rings”; ST14, “blue” rings; ST40, “purple” rings; ST45, “yellow” rings; ST48, “aquamarine” rings; ST147, “red” rings: ST392, “blue-green [teal]” rings; ST377, “pale green” rings; ST1308. The other undefined colored rings are STs that include one or two strains

Accessory genomes are those virulence markers that have variable presence [40]. Ferric uptake system, *kfuABC*, as an accessory genome was present in 9 (16.9%) strains, including ST1308, ST6510, high-risk clone ST14, and hvKp ST2159. Yersiniabactins, including “yersiniabactin receptor gene” (*fyuA*), *irp*1/2, and “yersiniabactin siderophore cluster” (*ybtAEPQSTUX*) were detected in 46.3% of strains and in association with 4 chromosomally “integrated conjugative elements” (ICEs), mainly from ST14, ST40, ST45, ST86, and ST392. The majority of yersiniabactin-carrying (*ybt*^+^) isolates, 22 out of 25 (88%), harbored ICE*Kp3* and ICE*Kp4* related to *ybt* 9 and *ybt* 1 lineages, corresponding to isolates from ST392/ ST86 and ST14/ST45/ST48, respectively. Other detected MGEs and *ybt* lineages with low frequency (< 5%) were *ybt* 16 /ICE*Kp12* and *ybt* 14 /ICE*Kp5* (Table 1). Aerobactin *iucABCD* (*iuc1*) and salmochelin (*iroBCDN*) (*iro1*) were detected among three hvkp strains, including two ST86 (positive for ICE*Kp*3) and one ST2159 strains (Fig. 4).

### Iranian ST147 strains are phylogenetically more related to European genomes

**Fig. 5:**
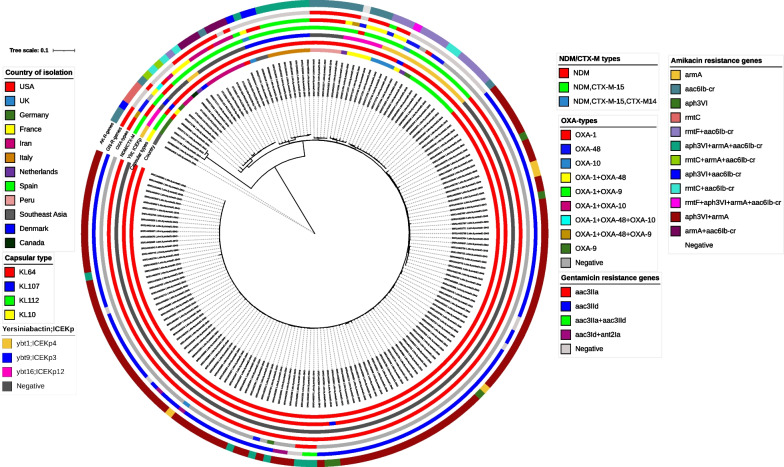
Maximum-likelihood phylogeny of ST147/NDM + strains based on whole genome SNPs analysis. A phylogenetic reconstruction of 183 strains (including the 174 downloaded genomes and our 9 study strains) with *K. pneumoniae* strain 4/1–2 (GenBank ID: CP023839.1, NDM + strain), as the reference strain is shown. Colored strips surrounding the phylogram represent the country of origin of each strain, capsular types, ICE types, carriage of NDM/CTX-M-15, OXA-types, and genes involved in gentamicin and amikacin resistance

By mapping 183 genomes of *K. pneumoniae*, including our nine ST147 strains and 174 publicly available ST147/NDM + genomes to *K. pneumoniae* strain 4/1–2 (GenBank ID: CP023839.1, NDM + strain), 2943 SNPs were identified. Isolates were clustered into different groups and the largest one was consisted of United States (Chicago region) genomes. The majority of downloaded genomes (n = 128) originated in the United States (except for two genomes; SRR3228444 and SRR8984905, all were from the Chicago region), followed by Europe (n = 36), Southeast of Asia (n = 4), South America (Peru; n = 5), and Canada (n = 1). The Iranian strains were clustered with three European/one Canadian (KL64/ O2v1) strains with ≤ 30 SNP differences (Fig. 5). Focusing on resistance markers revealed that the United States isolates harbor different pattern of these elements compared to other genomes, including *bla*_CTX-M-15_ (USA; 1.6% *vs*. 92.3%, *P* < 0.001), *bla*_OXA-48_ (USA; 0.8% *vs*. 41.5%, *P* < 0.001), *bla*_OXA-1_ (USA; 3.1% *vs*. 67.7%, *P* < 0.001), *bla*_OXA-10_ (USA; 0.8% *vs*. 12.3%, *P* < 0.001), *qnrS* (USA; 3.1% *vs*. 46.2%, *P* < 0.001), *armA* (USA; 93.8% *vs*. 26.2%, *P* < 0.001), *qnrB* (USA; 2.3% *vs*. 33.8%, *P* < 0.001), *qnrE* (USA; 0 *vs*. 7.7%, *P*: 0.004), *aac6Ib-cr* (USA; 6.3% *vs*. 84.5%, *P* < 0.001), *aac3Iia* (USA; 2.3% *vs*. 55.4%, *P* < 0.001), *rmtC* (USA; 1.6% *vs*. 10.8%, *P*: 0.007), *rmtF* (USA; 0 *vs*. 23.1%, *P* < 0.001), *aph3Via* (USA; 94.5% *vs*. 16.9%, *P* < 0.001), *bla*_OXA-9_ (USA; 0.8% *vs*. 13.8%), and *aac3IId* (USA; 93.8% *vs*. 0, *P* < 0.001). The link between the ST147 and the KL64 was found in this collection, except for nine strains that were identified as KL10, KL107, and KL112 (Fig. 5).

The comparable plasmid harboring *bla*_NDM-1_ was found in four downloaded genomes clustered with our strains, and IncF pMLST revealed it to be of the K2/K5:A22: B36 replicon type. Considering the ICE and Yersiniabactin types, all United States strains were negative. While 19 ICE*Kp*4 (*ybt* 1), nine ICE*Kp*12 (*ybt* 16) and 11 ICE*Kp*3 (*ybt* 9) positive isolates were identified. Genomic characteristics of ENA downloaded genomes have been shown in Additional file 2.

**Fig. 6:**
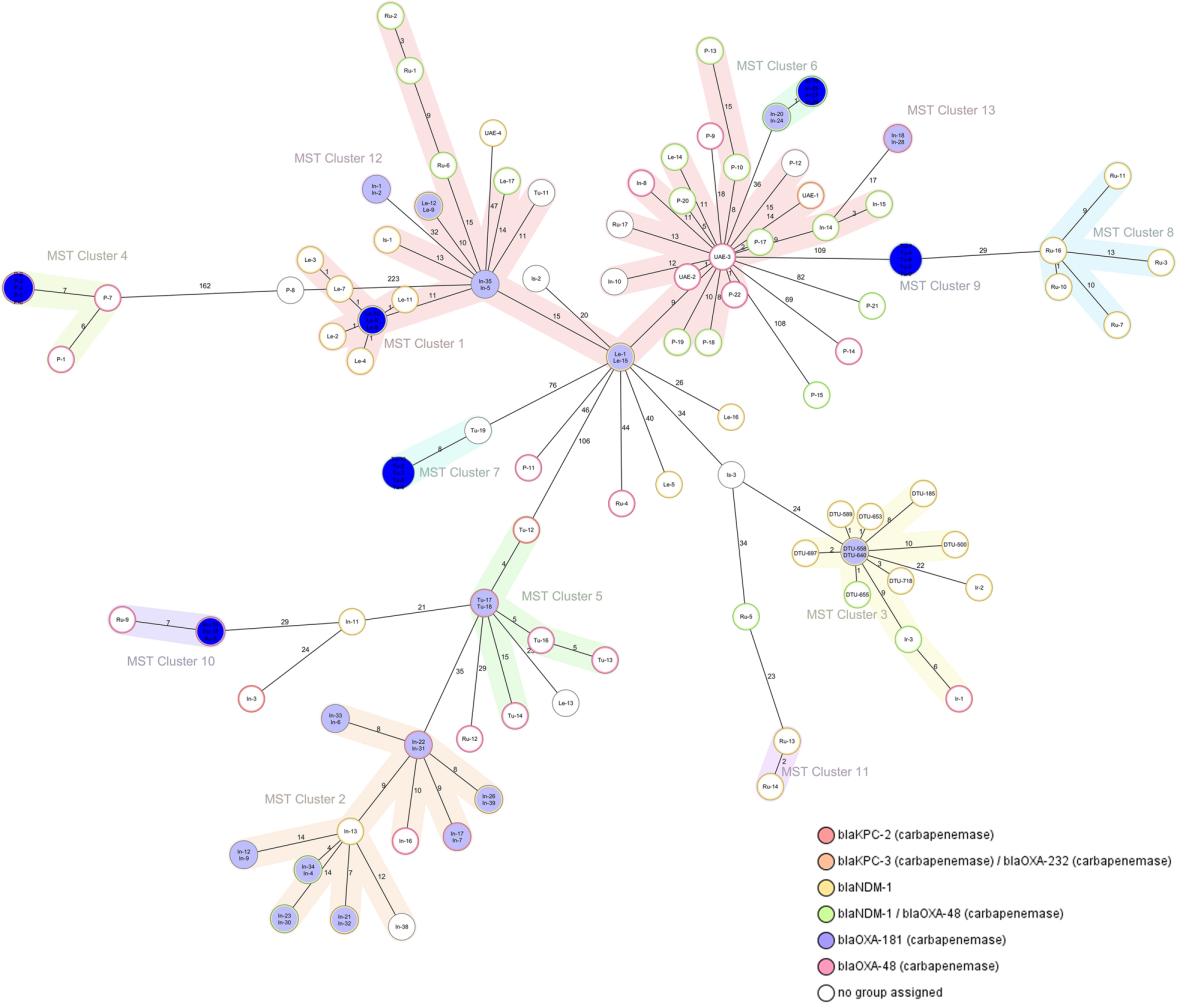
Minimum Spanning Tree showing distance based on cgMLST of 1826 genes using the parameter ‘pairwise ignoring missing values for 130 ST147 genomes. Each circle is named with the geographical origin, including DTU: our ST147 strains, Ir: Iran, Tu: Turkey, In: India, P: Pakistan, Is: Israel, Le: Lebanon, Ru: Russia, UAE: United Arab Emirates. The data of carbapenemase genes is shown by the coloured rings inside each bubble

ST147 *K. pneumoniae* genomes reported from neighboring countries in our geographic region were included in the cgMLST analysis to identify genetic relatedness. Using a ≤ 15 allele difference threshold in cgMLST analysis, strains were clustered in 13 complex types (CTs) and 18 singletons (Fig. 6). The CTs were formed by genomes from the same countries, except for one cluster (CT1) which was the biggest and included isolates from all countries. Iranian genomes (our 9 isolates and 3 previously reported) were grouped into cluster 3 (11 strains) and one strain remained as a singleton (Ir-2). Considering the clustered strains, the highest diversity was found among Indian strains that were categorized into 5 clusters, followed by genomes from Russia /Turkey (4 clusters) and Pakistan (2 clusters) (Fig. 6). Characteristics of downloaded genomes deposited from neighboring countries are shown in Additional file 3.

### Diversity of genetic context involved in spreading of *bla*_NDM-1_, *armA* and *bla*_CTX-M-15_

**Fig. 7:**
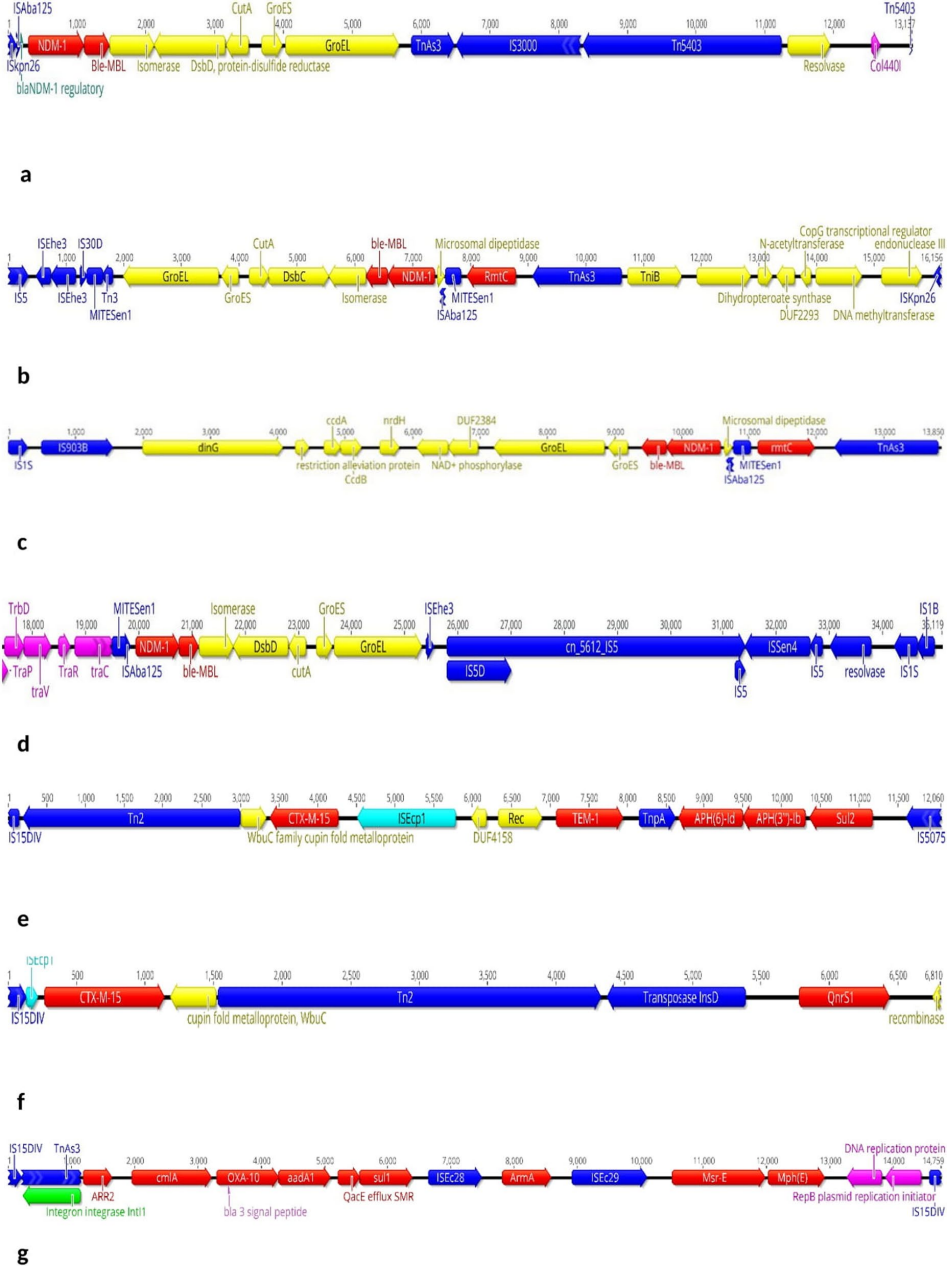
Genetic context of resistance markers among study population. **a** contig harbouring *bla*_NDM-1_ in ST392, **b**–**d** three forms of MGEs bracketing NDM-1, **e**, **f** two isoforms of MGEs arrangements in *bla*_CTX-M-15_ harbouring contigs, **g** the integron 1 cassette structure harbouring the *armA*

Analysis of the genetic context of *bla*_NDM-1_ in ST147 and ST392 as the positive clones corroborated the previous conserved sequence of this gene, showing that the NDM-1 gene is located downstream of truncated IS*Aba125* and is followed by *ble*_MBL_, Isomerase, protein disulfide reductase *dsbD*, *cutA*, and *GroES-GroEL* genes; however, there were some differences between these two clones based on the arrangement of mobile genetic elements (MGEs) surrounding this segment. Among ST392 strains, only one isoform of MGEs, including ΔIS*Kpn26*-NDM core structure-Tn*As3*-ΔIS*3000*-Tn*5403* was found on plasmid col440I (Fig. 7a). In ST147, the NDM-1 core structure was discovered upstream of the *rmtC* 16S rRNA methylase gene, flanked by MITE*Sen1* and IS*903B* in one strain and MITE*Sen1*/MITE*Sen1* composite transposon in the other (Fig. 7b, c). In *rmtC* negative ones, the NDM core structure was bracketed by MITE*Sen1*/IS*Sen4* (Fig. 7d). The plasmid type of NDM in ST147 was IncFIB (PB171).

The *bla*_CTX-M-15_ harbored by ST14, ST45, ST48, ST377 and all except one CC147 strain. The core segment was arranged by a truncated and/or intact form of IS*Ecp1* upstream of CTX-M-15, and the "WbuC family protein fold metalloprotein" gene was located downstream but in a reversed direction along with a copy of Tn*2*. This core structure was co-localized with (i) *bla*_TEM-1B_, *tnpA*, *aph(6)-Id*, *aph(3)-Ib* and *sul2* genes, which were bracketed by IS*15DIV* and IS*5075* (IS*110* family), and (ii) IS*2* and *qnrS1*. The length of CTX-M-15 carrying contigs varied across STs; however, data suggest a common origin via horizontal transfer across multiple sequence types. (Fig. 7e, f).

The other *16S* rRNA methylase gene, *armA*, was detected in seven out of nine ST147 and one ST48 strain (no. 639). The core structure which was located on the class 1 integron structure was consisted of *armA* bracketed by IS*Ec28* and IS*Ec29*, along with *ARR-2*, *cmlA1*, *bla*_OXA-10_, *aadA1*, *qacE*, and *sul1* in downstream, and *msr(E)/mph(E)* in upstream. The genetic environment of this structure was conserved in all positive strains and was flanked by two copies of IS*15DIV* (Fig. 7g).

## Discussion

We have identified different lineages of carbapenem non-susceptible *K. pneumoniae* and notably six carbapenemase (*bla*_OXA-48_, *bla*_NDM-1_, and no *bla*_KPC_) producing clones in our clinical setting, suggesting multiple importation events of this resistant pathogen into the hospital along with their clonal expansion at different levels. While CC147 was the largest clone with the greatest number of resistance markers, its two SLV members (ST147, ST392) varied in their carbapenem MIC values and resistance marker repertoires, with ST147 dominating. To assess the dissemination of *bla*_OXA-48_/*bla*_NDM-1_, we focused on the identification of MGEs, and two types of NDM-1 carrying plasmids ([IncFIB(pB171) exclusively identified in ST147 and Col440I mostly among ST392]) were identified. Furthermore, the IncL/M plasmid was exclusively detected among OXA-48 producers, and we could predict its location on this plasmid type. Different types of NDM-bearing plasmids with distinct dissemination patterns among two members of a clonal complex appear to be largely dependent on the plasmid backbone and the bacterial sequence type harboring them. Our effort in transconjugation assay was successful in transferring of *bla*_NDM-1_ carried by two different replicon types, highlighting the important role of these molecular vehicles in NDM circulation. On the other hand, the plasmids harboring *bla*_OXA-48_ did not transfer successfully. Maybe it is due to a low efficiency of their conjugation trait during transcojugation assays. However, it seems that the plasmids carrying *bla*_NDM-1_ can easily transfer and expand the antimicrobial resistance among inter- and intra-species of hospital-resident bacteria. In a recent study of carbapenemase producing *Enterobacterales* collected from a university hospital in Tehran, Iran, the IncFII and IncA/C plasmids were identified as the vehicles for carriage of *bla*_NDM-1_ in *K. pneumoniae*/*Enterobacter cloacea* and *E. coli*, respectively [13]. Nevertheless, other types of plasmids such as IncL/M, IncFIB(pQil), IncFII (PRSB107) and IncFIB/HI1B hybrid plasmid are also reported for NDM circulation [3, 41], indicating that independent acquisition of NDM is more important than its widespread dissemination via major transferable plasmids [42].

The genetic environment surrounding the NDM-1 segment (IS*Aba125*-*bla*_NDM-1_-*ble*_MBL_-Isomerase-*DsbD*-*cutA*-*groEL*/*ES*) was different between CC147 members as well. While MGEs arrangement appeared to be conserved in ST392 strains (flanked by ΔIS*kpn26* and Tn*5403*), that was completely divergent among ST147, consisting of different patterns of MITE*Sen1* composite transposon, IS*Sen4*, MITE*Sen1*, IS*Ehe3*, IS*5*, and IS*1*. This rearrangement diversity of MGEs surrounding NDM in a single circulating clone of *Klebsiella,* with strong evidence of homogeneity based on genomic analysis and the sampled geographic region, was intriguing. This finding indicates that *bla*_NDM-1_ is embedded in dynamic genetic platforms. From a clinical point of view, spreading of *bla*_NDM_/*rmtC*/*armA* co-producing ST147 clone is worrying, as this resistance combination limits the effectiveness of combined carbapenem-aminoglycoside therapy in severe infections.

Genome-wide analysis of study CC147 revealed a tight cluster with < 15 SNPs among ST147, while ST392 strains were more divergent based on genetic context (≈ 50 SNPs difference) and resistance/virulence repertoire. Incorporating the isolation dates of NDM-producers during the sampling period revealed that the NDM gene was first circulated in 2018 by ST392 and gradually replaced by ST147, which was more homogeneous and harbored the most robust resistance repertoire to spread this carbapenemase. It has been shown that *K. pneumoniae* propensity for successful colonization of hospital environments is strongly influenced by the resistance level, and that carbapenemase producers are prioritized in this regard [42]. The probable explanation might be that ecological constraints exerted by antibiotic exposure obstruct the spread of isolates with a lower capacity for expressing resistance [43]. Constructing a phylogenetic tree using SNP-based and cgMLST approaches that included our ST147 and publicly available corresponding genomes revealed a monophyletic cluster of our nine strains, indicating a single regional introduction, probably through a locally circulating ST147 strain. Furthermore, the IncF-plasmid replicon found in our ST147 was also detected in four downloaded genomes co-clustered with our strains, suggesting that the IncF plasmid we reported here [IncFIB (pB171)] can be stably associated with a high-risk ST147 clone.

During the search for NDM dissemination in our setting, the ICU was identified as the likely source. There are some points that likely play roles in the spread of NDM-producing CC147. Patients in the ICU are at high risk of acquiring carbapenemase-producing Enterobacteriaceae (CPE), and long-term ICU stays increase the risk of acquiring multidrug-resistant organisms [44]. Furthermore, multibed rooms in internal wards connected to ICUs via patient transfer are common, and the facilities themselves lack resources for infection control approaches, both of which could contribute to the intra-facility spread of MDR-high risk clones [45].

Whereas a high diversity of K loci exists, few O loci are described in this organism, with O1, O2 and O3 loci being mostly associated with human disease [46]. Two distinct O loci of LPS, O1/O2v1 loci were predominantly found in urine, blood and respiratory origin isolates. O1/O2v1 loci have been described in association with invasive tissue infection and colonization of internal organs [47]. So, these isolation sites and the subtypes of recovered strains should not be undervalued, since they could result from intestinal colonization as an initial step for progression to disease.

Despite the conserved nature of O and K-locus antigens identified within our genomes, the virulence gene content differed even within the study clones. It was highlighted in the ST392 lineage, where half of them were positive for ICE*Kp3*, *mrkA-H* type 3 “fimberiae synthesis cluster”, and a complete *fyuA*-*irp*-*ybt* “yersiniabactin biosynthetic operon”. The lowest prevalence of virulence markers was the ferric uptake system (*kfuABC*) that harbored by 16.9% of isolates, including K1/ST2159 hvKp and ST14 MDR/high-risk clone. Higher rates of siderophore production have been proposed to be associated with hvKp lineages and hypermucoviscosity phenotype. However, this measurement has been shown to be only partially correlated with in vivo virulence differences [48]. Conversely, it has been revealed that CC258/KPC^+^ harboring yersiniabactin shows an enhanced ability to colonize the respiratory tract and cause pneumonia in experimental infection models [49]. Overall, it appears that co-carriage of ICEs/ yersiniabactins with carbapenemases/ ESBLs (CC147, ST14, ST45, ST48 and ST377, in our study) is concerning, as this phenomenon not only could be associated with infections but also be a frequent first step in the acquisition of more siderophores (specifically plasmidic siderophores *iucABCD*/*iroBCDN* operons considered for hvKp) that enhance gut colonization ability of KpI population and consequently invasive resistant infections with high mortality rates [50].

We acknowledge the limitations of our survey. Firstly, the study was conducted in a single acute-care referral hospital in Iran and the sampling period was short, so re-running this survey along with *bla*_NDM_ producing strains collected from other centers will be necessary to confirm current findings. Second, the patient's medical history (specifically antibiotic usage and the hospitalization period before and after of CC147 isolation) was missing, so we couldn’t find any link between our phylogenetic data and epidemiological information. Thirdly, the number of deposited public genomes that we analyzed alongside our phylogeny was limited, and we will need to include more ST147 genomes mostly that originated from our geographical region, specifically, Persian Gulf countries, India and Pakistan, to gain a better understanding of how this dominant carbapenemase-producing clone circulates.

## Conclusions

The spread of circulating CC147 super-clone with high genomic plasticity in the acquisition of various resistance/virulence markers that propagate along transmission poses a significant threat to public health. The highly convergent nature of CC147 with different subpopulations is linked to specific genomic features and geographic distribution [8]. With the help of different arrangements of MGEs, subpopulations of a single clonal complex bring notorious resistance markers such as NDM into circulation together, as we have highlighted. The public health sector should therefore prioritize genomic surveillance of pathogens, detect the introduction and spread of high-risk clones early in an epidemic, and strengthen the resilience of national hospital referral networks to prevent the spread of infectious diseases.

### Supplementary Information

**Additional file 1: Figure S1.** The sunburst plot displaying proportions of horizontally acquired resistance markers (presented as percentage) which provide resistant phenotype against antibiotics other than b-lactams. The gene *aac(6)-Ib-cr* has been shown in association with two antibiotic families, Aminoglycosides and Fluoroquinolones, as this element confers resistance to both groups. Diagram was generated with the ggplot2 using software R 3.0.1.

**Additional file 2.** Genomic characteristics of ENA.

**Additional file 3.** Characteristics of ST147 K. *pneumoniae* genomes deposited from Iran and countries in our geographic region.

## Data Availability

The raw sequence data supporting the conclusions of this article is available in the European Nucleotide Archive (https://www.ebi.ac.uk/ena/browser/home) under study accession number PRJEB59975.
